# Scottish National Complex Renal Cyst Surveillance Protocol

**DOI:** 10.1002/bco2.70094

**Published:** 2025-10-08

**Authors:** Benjamin Parkin, Gavin Lamb, Nikolas Arestis, Anna Brown, Zack Slevin, Jane Hendry, Steve Leung, Julian Y. Keanie, James Blackmur, Sara Ramsey, Ross N. Clark, Abdel Hamed

**Affiliations:** ^1^ NHS Forth Valley Stirling UK; ^2^ NHS Greater Glasgow and Clyde Glasgow UK; ^3^ NHS Lothian Edinburgh UK; ^4^ NHS Highland Inverness UK; ^5^ NHS Ayrshire and Arran UK

**Keywords:** Bosniak classification, Bosniak cyst, Bosniak IIF, Bosniak IIF follow‐up, complex renal cyst

## Abstract

**Introduction:**

Aim to design and test a suitable risk‐targeted imaging protocol for follow‐up of complex renal cysts categorised IIF.

**Patients and Methods:**

The Scottish Protocol was designed at a joint meeting with the Scottish Urological and Scottish Radiological societies according to published data on imaging modality, classification criteria and interval progression of Bosniak IIF renal cysts. Patients were listed prospectively to follow this protocol across five NHS health boards within Scotland. Patient data accessed between Aug 21 and Feb 22. All patients with a confirmed Bosniak IIF cyst on computerised tomography or magnetic resonance imaging after multi‐disciplinary team review were included. Patients were reviewed according to progression, interval, treatment and histology.

**Results:**

A total of 160 patients were identified with Bosniak IIF cysts. 98 (61%) were male (age range 29–97, median 67, IQR 57–75). Thirty‐four patients completed the proposed 4‐year follow‐up. Seventeen patients advanced to treatment, with 15 patients having confirmed malignancy (9.4% of the total database). The mean time from diagnosis to intervention was 1 year and 2 months (range 34 to 1172 days). No patients developed metastatic disease during follow‐up.

**Conclusions:**

The Scottish Complex Renal Cyst Protocol provides a risk‐targeted imaging framework that reliably identifies patients with progressive lesions prior to the development of advanced disease. Incidence of progression is consistent with published data of 9.4% most commonly within 2 years, and not beyond 4 years of surveillance.

**Aims**

- To determine whether the Scottish protocol for follow‐up of complex renal cysts is effective in identifying advancing disease requiring treatment
- Ensure patients do not progress beyond treatment during follow‐up
- To determine an appropriate length of follow‐up for complex renal cysts in Scotland
- Findings are in keeping with Bosniak's research, suggesting that 5–8% of patients with Bosniak IIF cysts develop into malignant disease

## 1 INTRODUCTION

The Bosniak classification of renal cysts was first proposed in 1986, with distinguished categories I to IV.^1^, ^2^ The aim of the classification system was to evaluate complex renal cysts and aid the clinical management of such.^3^

The classification system was updated in 1993 to include a bridging category between Bosniak II and III, deemed Bosniak IIF (F for follow‐up). These are thought to likely be benign, but due to their complexity, require follow‐up studies to ensure no progression.^4^, ^5^, ^6^, ^7^, ^8^ The true prevalence of malignancy within Bosniak IIF cysts is not known, but a strong indicator of malignancy is its progression over time.^9^ Schoots et al studied 954 stable Bosniak IIF cysts, and of the 54 resected, 9 (17%) were found to be malignant. 73 (11%) of Bosniak IIF cysts progressed to Bosniak III or IV, and of those, 85% were found to be malignant.^3^ Further studies have found similar results.^10^

Bosniak proposed that if the follow‐up approach is taken, the first follow‐up should occur 6 months following initial diagnosis – if the cyst remains unchanged, additional follow‐up should occur yearly for at least 5 years. At this time, it was not clear what the optimal follow‐up period would be, recognising that younger patients or more complex cysts may require longer follow‐up periods due to limited long‐term research regarding these patient groups.^6^

It has previously been noted that the initial imaging modality may affect the initial classification of a Bosniak cyst, with MRI likely leading to upward classification when compared to CT.^11^, ^12^, ^13^ There has also been evidence to show there is class assignment variation between radiologists.^14^, ^15^ It has been noted that more experience does help with this issue,^2^ however, classification of Bosniak II, IIF and III cystic lesions varied significantly.^15^

Further evidence supports that most lesions that demonstrate signs of progression do so within the first two years of surveillance and rarely beyond 4 years of being identified.^9^ Previous follow‐up guidance has not reflected this, and within Scotland, it was noted that there was significant variation in the follow‐up interval and duration of Bosniak IIF cysts. To try and address these variations, a joint meeting of the Scottish Radiological Society and the Scottish Urological Society was held in November 2015 in the wake of the emerging data. This produced a consensus agreement that a consistent follow‐up protocol for complex (Bosniak IIF) renal cysts was required. This was aimed at strategising the administration of ionising radiation appropriately and focusing follow‐up at the point of greatest risk of progression and managing resources appropriately. Seminar presentation, literature review and round table discussion agreed upon the Scottish Complex Renal Cyst Follow‐up Protocol (Appendix S1). The protocol was designed to establish consistency of reporting with radiology review at uro‐radiology MDT, reduce radiation exposure utilising a single portovenous phase follow‐up, target scanning to the first 2 years and allow 4‐year follow‐up with the option of extension in younger or higher risk patients.

There was a clear consensus in Scotland that Bosniak IIF status should be done by an initial triple‐phase computerised tomography (CT) or contrast‐enhanced MRI. Standard follow‐up imaging was agreed as CT surveillance at 6 months, 18 months, 30 months and 48 months using single post‐contrast (nephrographic) phase CT. MRI could be used in patients <50 years depending on resource availability. Patients may be discharged if no change at 4 years, with the option of extended follow‐up in younger or high‐risk patients. We aimed to show that this protocol was effective in real‐world clinical settings.

## 2 METHODS

Patients were collected from five NHS health boards throughout Scotland; these included NHS Ayrshire and Arran, NHS Forth Valley, NHS Greater Glasgow and Clyde, NHS Highlands and NHS Lothian between September 2015 and September 2021.

All patients with a radiologically confirmed Bosniak IIF cyst were included in the study. If the investigation confirming diagnosis was not performed by the urology department, these patients were referred to urology directly from radiology as per the agreed protocol to ensure that appropriate follow‐up was arranged. Scans were reviewed by specialist uro‐radiologists, allowing the opportunity for reclassification as required at diagnosis. An appointed clinician collected patient data information within each health board, and this data was re‐analysed to ensure accuracy.

Each community health index (CHI) number was provided, which allowed for their clinical information to be accessed through the clinical portal – the digital documentation hub. Each patient was analysed with regard to their initial presentation, time from diagnostic scan to triple phase CT (if not their diagnostic scan), CT/MRI scan, clinic appointment letters and further presentations to the hospital post follow‐up. Any patient who died had their final admission reviewed, if available, as well as their death certificate.

Initial scans were analysed for the presence of enhancement and calcification, the number of septations, the maximum thickness of septations and the maximum cyst size. These factors were then re‐analysed with follow‐up scans to determine if there had been any changes. If changes were present, then scans would be re‐discussed at MDT meetings to decide whether to continue follow‐up or reclassify the cyst and alter the management plan.

Patients advancing to treatment were further analysed regarding their trigger to treatment, their surgical modality, time from diagnosis to treatment and whether they had malignant pathology. All patients advancing to treatment had their scan requests reviewed to ensure they were in line with the Scottish protocol.

**FIGURE 1:**
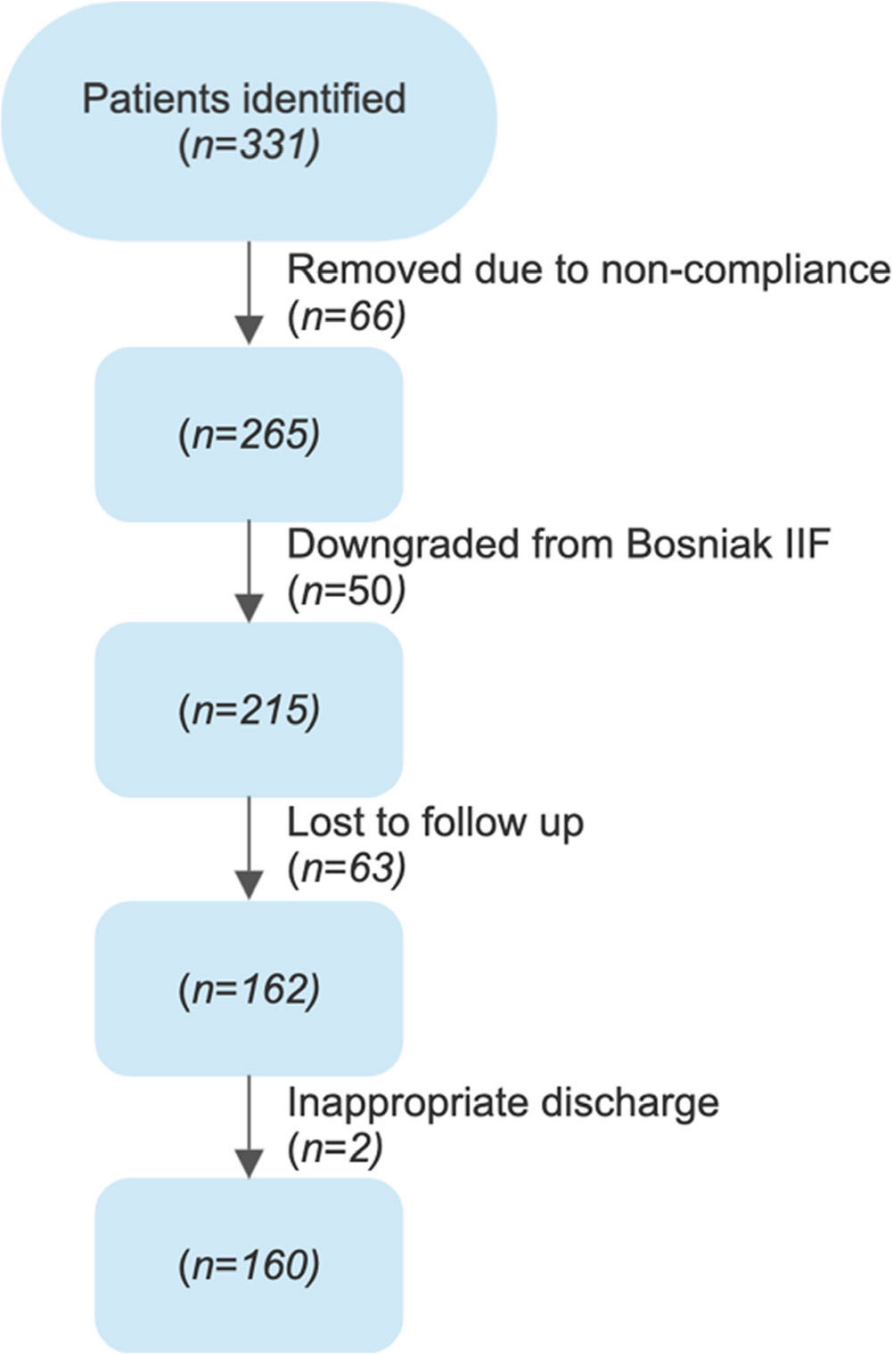
Flow diagram detailing exclusion criteria and patient selection for analysis.

A total of 331 patients were initially added to the study. A number of patients were downgraded or upgraded at primary review, and they were excluded from this study prior to the initial count. A total of 63 patients were lost to follow‐up due to moving out with the health board or not attending for their radiology appointments. Throughout the follow‐up period, 50 patients were downgraded from Bosniak IIF to Bosniak II and did not require any further follow‐up. A total of 66 patients were removed due to non‐compliance with the protocol, or their initial scans were conducted prior to 15/11/2015. Two patients were inappropriately discharged from the protocol early. Therefore, 160 patients were followed up according to the Scottish Protocol and analysed within the study (See Figure 1). A 10‐month ‘grace period’ was granted for each scan interval due to the Covid‐19 pandemic.

## 3 RESULTS

The age range of patients was 29–92 (median = 67, IQR 57–75). A total of 98 were male (61%) and 62 were female (39%). Initial referral included 115 (72%) with incidental findings, 27 (17%) with haematuria and the remaining 18 were investigated for a variety of reasons, including flank pain and dysuria. Patients were first identified using CT scan (*n* = 92, 57.5%), ultrasound (*n* = 64, 40%) and MRI (*n* = 4, 2.5%). Patients with primary imaging ultrasound or single‐phase CT were further imaged with triple‐phase CT prior to MDT validation review.

A total of 34 patients (21.3%) had fully completed the Scottish follow‐up protocol, with 21 patients (61.8%) being discharged at 4 years; a remaining 12 patients (35.3%) were maintained for 1 further scan due to their young age, and 1 died of unrelated causes. The remaining patients are awaiting further investigations, with some delayed in the protocol due to the Covid‐19 pandemic – all patients were retained within the study despite these delays. The compliance rate, within this group, for following the Scottish protocol was 97.1% (33 patients).

A total of 17 patients (10.6%) advanced to receiving treatment. The trigger for treatment varied, including upgrade to Bosniak III (*n* = 10, 58.8%), upgrade to Bosniak IV (*n* = 4, 23.5%), confirmed malignant pathology from biopsy (*n* = 1, 5.9%) and high suspicion of malignancy (*n* = 2, 11.8%). One patient is currently awaiting treatment after being referred for cryoblation. The average time from initial diagnosis to intervention was 442 days (median: 390 days, range: 34–1172 days). The surgical modality varied between patients, dependent on their cyst complexity, co‐morbidities and suitability for minimally invasive approaches (Table 1).

**TABLE 1:** Surgical approaches for all patients undergoing treatment within the Scottish protocol.

Surgical approach	*n*=	Percentage (%)
Laparoscopic nephrectomy	7	41.2
Robotic laparoscopic partial nephrectomy	2	11.8
Open nephrectomy	3	17.6
Open partial nephrectomy	2	11.8
Cryoblation	3	17.6

Fifteen patients were found to have malignant pathology (88.2%) – 9.4% of the studied database (Table 2). The other two patients did not have pathology results; they underwent cryoblation. Follow‐up has demonstrated no metastatic disease. Of the patients advancing to treatment, two of them have since died, and both deaths were unrelated to their Bosniak IIF cyst or surgery.

**TABLE 2:** Pathology results for all patients undergoing treatment within the Scottish protocol.

Pathology	*n*=	Percentage (%)
Papillary renal cell carcinoma	10	58.8
Multilocular cystic renal neoplasm of low malignant potential	5	29.4
Unknown	2	11.8

Of the initial 331 patients studied, there have not been any reported cases of incurable disease related to their Bosniak IIF cyst. All patients undergoing treatment were cured of their disease, and the remaining 20 living patients have either been discharged or are undergoing annual follow‐up as per the Scottish guidelines.

## 4 DISCUSSION

In this multicentre, real‐world review of a follow‐up protocol for patients with Bosniak IIF cysts, we have shown 10.6% (*n* = 17) of patients advancing to treatment, and 15 (88.2%) of these patients being confirmed to have malignant pathology (while the other 2 underwent cryotherapy with no preceding biopsy). A total of 9.4% of the total database was found to have developed malignant disease.

There are few studies that demonstrate longitudinal follow‐up of Bosniak IIF cysts, particularly within the United Kingdom, as protocols vary significantly throughout the world and within the United Kingdom itself. Previous studies have demonstrated that progression to malignancy ranges from 7% to 15%,^16^, ^17^, ^18^, ^19^ and that this is a significant portion of those with a diagnosed Bosniak IIF cyst. Findings within our results support this, with Bosniak IIF cysts are often found incidentally during abdominal CT or ultrasound scans, with initial presentation being varied. Accurate diagnosis of Bosniak IIF cysts remains a difficulty within diagnostic radiology.^2^, ^8^, ^20^ Bosniak IIF cysts are likely to be benign but have the potential to differentiate into malignancy and therefore require follow‐up studies in order to demonstrate stability. Those that show slow progression and show development in septations, calcification or enhancement are likely to be Bosniak III or IV and therefore require surgical intervention.

Bosniak suggested that a 4‐year follow‐up period would likely be sufficient, and following discussion in the joint meeting of the Scottish Radiological Society and the Scottish Urological Society held in November 2015, it was felt that a 4‐year follow‐up approach would be most appropriate. Our study has shown that the time between initial diagnosis and treatment averaged 442 days (~1 year and 2 months), ranging from 34 to 1172 days (~3 years and 2 months). Any patient with changes to their cyst at the final scan or who was of a young age (<50 years) was not discharged and would undergo a final scan at 5 years to determine whether they were fit for discharge. To our knowledge, no patient that completed follow‐up has since developed metastatic disease.

The imaging features that determine whether a Bosniak IIF cyst has progressed are based on whether there has been an increase in the thickness of septa, whether enhanced solid areas have appeared, an increase in the number of irregularly enhanced septa, or the thickness of the enhanced wall.^19^ Unfortunately, this study was unable to identify whether any of these factors played a more significant role than the others due to the small sample size of patients advancing to treatment. The study will continue to track all remaining patients until they have completed their follow‐up period, and the results will be re‐analysed.

It must be recognised that 50 patients (28.1%) were downgraded from a Bosniak IIF to either a Bosniak I or II cystic lesion – with the majority of these occurring following the 6 monthly scan. All patients had undergone MDT review of their triple‐phase imaging and were felt to be indeterminate; therefore, further supporting the use of the Scottish Protocol.

Limitations are acknowledged in this study. Whilst patients were identified prospectively to follow the Scottish protocol, this is a retrospective audit from multiple health boards throughout Scotland, with some patients still undergoing their follow‐up. Each health board dataset was collected by different individuals, which may lead to inclusion bias or misinterpretation of scan results. To resolve this issue, all data were then re‐checked by one individual to ensure accuracy, but again, it remains open to misinterpretation of scan results and clinic letters.

Initial radiological diagnosis is open to interobserver bias and ranges in experience and training between clinicians. All patients with a suspected Bosniak IIF cyst were discussed at MDT, and some were downgraded at this stage to Bosniak I or II and therefore did not require follow‐up. Going through the MDT ensures that individual bias or experience is removed and a uniform decision is made.

Whilst 9.4% of Bosniak IIF cysts within our study progressed to malignancy within 4 years, there is no certainty that those discharged would not develop malignant disease in the years following discharge, as cystic lesions typically progress slowly.^19^ We know that malignancy can occur within benign appearing cystic lesions, but the timeframe in which this progression happens is not always clear.^19^, ^21^, ^22^ Our study has shown that a follow‐up period of 4 years should be sufficient in identifying progressive lesions, with the exception that younger patients may have an extended follow‐up of 5–6 years. Our findings are in keeping with similar studies.^3^, ^10^, ^19^

In conclusion, based on the results collected from five NHS health boards within Scotland, 9.4% of Bosniak IIF cysts progressed to malignancy. The Scottish Protocol reliably identified patients using a rationalised and targeted schedule, allowing timely treatment and managing both radiation exposure and clinical resources effectively. Therefore, the recommendation is to follow‐up patients for at least 4 years with the interval scans as outlined, with longer follow‐up periods being appropriate in young patients and those with slight changes in their cysts over a prolonged period.

## 5 RECOMMENDATIONS

The Scottish protocol for follow‐up of complex renal cysts has been shown to be effective in the diagnosis of malignancy in Bosniak IIF cysts. The suggested follow‐up of 4 years has been shown to be necessary, with one patient undergoing surgery at 3 years and 2 months following their initial diagnosis.

It is recommended that all trusts within Scotland follow this uniform protocol (Appendix 1).

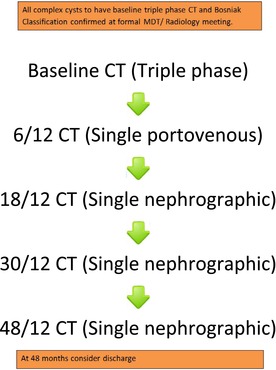

## AUTHOR CONTRIBUTIONS

- Parkin, B – Lead author Forth Valley
- Lamb, G – Secondary author Forth Valley
- Arestis, N – Secondary author Forth Valley
- Brown, A – Data colleciton Greater Glasgow and Clyde
- Slevin, Z – Data collection Forth Valley
- Hendry, J – Data collection Greater Glasgow and Clyde
- Dardis, A – Data collection Greater Glasgow and Clyde
- Looi Wan Wen, C – Data collection Greater Glasgow and Clyde
- Leung, S – Data collection Lothian
- Keanie, J – Data collection Lothian
- Blackmur, J – Data collection Lothian
- Ramsey, S – Data collection Highland
- Clark, R – Data collection Ayrshire and Arran
- Hamed, A – Data collection Ayrshire and Arran

## CONFLICT OF INTEREST STATEMENT

No conflicts exist.

## Supporting information


**Appendix S1.** Supporting Information.
