# Supplementary material for: Scottish National Complex Renal Cyst Surveillance Protocol
Source: BJUI Compass. 2025 Oct 8;6(10):e70094. doi: 10.1002/bco2.70094 (PMC12504850; doi:10.1002/bco2.70094)
Supplement: Supplementary file 1 — Appendix S1. Supporting Information. [file BCO2-6-e70094-s001.docx]

Appendix 1

Scottish National Bosniak 2F Follow Up Protocol


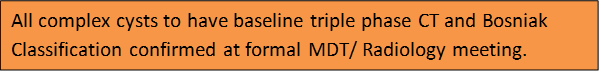


Baseline CT (Triple phase)


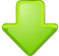


6/12 CT (Single portovenous)


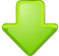


18/12 CT (Single nephrographic)


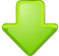


30/12 CT (Single nephrographic)


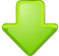


48/12 CT (Single nephrographic)


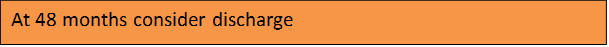


*Qualified intervals are in months from baseline CT
